# Honey: Chemical Composition, Stability and Authenticity

**DOI:** 10.3390/foods8110577

**Published:** 2019-11-15

**Authors:** Olga Escuredo, M. Carmen Seijo

**Affiliations:** Department of Vegetal Biology and Soil Science, Faculty of Science, University of Vigo, As Lagoas, 32004 Ourense, Spain; mcoello@uvigo.es

**Keywords:** botanical origin, physicochemical characteristics, geographical origin, biological properties, chemometrics

## Abstract

This Special Issue contains innovative research papers on the characterization, chemical composition and physical properties of honey. This constitutes very useful information to avoid frauds and to guarantee the authenticity of this food product. The knowledge of the particularities of honey is increasingly demanded by beekeepers and consumers, and also by labs to typify honeys according to their botanical origin and to check their quality. Melissopalynological, sensorial and physicochemical techniques are being used to study the characteristics of honeys samples from different plant sources and geographical areas. The combination of these analytical techniques with mathematical and statistical methods or chemometrics allows researchers to identify a set of variables or individual parameters that define independent samples, providing a practical solution to classify honey according to the geographical or the botanical origin.

Honey is obtained from the nectar of plants, plant secretions or excretions mediated by sucking insects, such as aphids, or from blends of them [1,2]. The predominance of one of these sources leads classification of honey as either blossom honey or honeydew honey [1,3]. Honey has a very complex composition, which depends on the botanical and geographical origin, the weather during harvest, the climate conditions of the area and the apicultural management, especially during the honey harvest and storage [4,5,6,7]. 

Authentication of honey is a matter of great interest worldwide since it involves producers, sellers, exporters, control labs and consumers. It is especially useful to avoid frauds derived from adulterated or false-labeled honeys, which may enter the global market [8]. A good quality of honey and the proper labelling according to the botanical and geographical origin of the product are the main demand of honey consumers, who request the safety and the guarantee label of foods of their daily diet. Some quality parameters (such as water content, pH, electrical conductivity, HMF content, diastase activity or reducing sugars) inform about the nectary origin of the product and confirm the hygiene conditions for the manipulation and storage of honey [6,9]. Unifloral honey authentication requires different analytical techniques including pollen analysis, statistical evaluation of physicochemical data and identification of chemical markers by chromatographic techniques [10]. Hence, the chemical composition, stability and authenticity of honey collaborate in the integration of the quality of this product.

In addition to this, honey stands out as a healthy natural food, traditionally used as an alimentary supplement to which are attributed many antioxidants and antimicrobial properties [1,3,4,5]. The botanical origin is directly related with these properties; therefore, a good typification of honey is necessary. One of the relevant issues to be considered during the characterization of the product is knowledge concerning how bees produce honey and how different environmental and human factors can affect the honey properties. Hence, the standardization of unifloral honeys remains a demanded task. All this complemented with the use of new technologies or combined with traditional technologies could offer real security when labelling and marketing different honey types. We hope that researches such as that included in this Special Issue contributes the spread of rigorous scientific information on unifloral honey produced worldwide. 

In this Special Issue, we aim to publish research papers on physicochemical characteristics, sensorial characteristics, botanical origin identification, nutritional value, functional properties, antimicrobial food compounds, health compounds, and food authenticity and adulteration. Papers selected for this Special Issue were subject to a rigorous peer review procedure, with the aim of rapid and wide dissemination of research results, developments, and application in the honey industry.

The objectives of the papers published were as follows:

The paper presented by Karabagias et al. [7] evaluated the quality and bio-functional properties of honeys collected in Portugal. Considering the pollen profile and physicochemical characteristics (°Brix, moisture content, pH, electrical conductivity, free acidity, total dissolved solids, salinity, vitamin C content, specific weight, bio-activity parameter, volatile compounds, and color-metrics) the samples were classified as unifloral honeys of eucalyptus, chestnut and heather. In addition, some of these parameters were highly correlated using bivariate statistics. According to the authors, using a multi-parameter analysis, Portuguese honeys could be characterized by some sensorial characteristics such as color and aroma.

The main purpose of study presented by Karabagias and Karabournioti [8] was to differentiate Egyptian clover and citrus honeys using a set of simple and reproducible tests for physicochemical parameters in combination with supervised statistical tools. Linear discriminant analysis showed that eight physicochemical parameters (color coordinates of the CIELAB scale, total dissolved solids, salinity, moisture, free acidity, total acidity, and total dissolved solids/total acidity ratio) classified the honeys according to floral origin. The classification rate was of 95.5% using the original method and 90.9% using the cross-validation method. Specific conventional physicochemical parameters and color parameters in combination with chemometrics techniques had the potential to enhance the differences in floral Egyptian honeys.

Seijo et al. [2] investigated the physicochemical and palynological characteristics of oak honeydew (*Quercus pyrenaica* Willd.) honey and evergreen honeydew (*Quercus ilex* L.) honey produced in Spain. In recent years, honeydew honeys have developed great commercial demand due to the biological qualities they contain. In this paper, a multivariate analysis using the most representative pollen types and physicochemical components facilitated the differentiation of the honeydew honey samples from different source and geographical origin. Despite being honeys with similar sensorial and physicochemical characteristics, the combination of physicochemical and melissopalynological data with appropriate statistical techniques can be useful to honey characterization.

The work presented by Escuredo et al. [11] focuses on an extensive study of the chromatic characteristics of color by the CIELAB scale and the Pfund scale of unifloral honeys and honeydew honeys from Northwest Spain. A pattern on the color scale based on instrumental methods was characterized for chestnut, heather, eucalyptus and honeydew honeys. CIELAB coordinates were the parameters that best differentiate the different types of honey. Heather honey was a dark amber honey with reddish tonalities, while eucalyptus and blackberry honey presented a similar amber color value with higher values of hue (more yellowish honeys). Multivariate statistical techniques were used to evaluate the influence of the particular botanical origin and physicochemical parameters in color. It was possible to predict some color variables (Pfund, chroma and hue) with a multiple linear regression analysis including electrical conductivity, pH, flavonoid, and polyphenol content and the main pollen types of honeys.
